# Design, Characterization, and Wound-Healing Evaluation of Sodium Humate Transferosome-Loaded Alginate/HPMC Dermal Patches

**DOI:** 10.3390/pharmaceutics18030290

**Published:** 2026-02-27

**Authors:** Plamen Katsarov, Plamen Simeonov, Elisaveta Apostolova, Yana Gvozdeva, Radka Boyuklieva, Paolina Lukova, Ilia Bivolarski, Maria Koleva, Cédric Delattre, Vesela Kokova

**Affiliations:** 1 Department of Pharmaceutical Technology and Biopharmacy, Faculty of Pharmacy, Medical University of Plovdiv, 4002 Plovdiv, Bulgaria; plamen.simeonov@mu-plovdiv.bg (P.S.); yana.gvozdeva@mu-plovdiv.bg (Y.G.); radka.boyuklieva@mu-plovdiv.bg (R.B.); 2 Research Institute at Medical University of Plovdiv (RIMU), 4002 Plovdiv, Bulgaria; elisaveta.apostolova@mu-plovdiv.bg (E.A.); vesela.kokova@mu-plovdiv.bg (V.K.); 3 Department of Pharmacology, Toxicology and Pharmacotherapy, Faculty of Pharmacy, Medical University of Plovdiv, 4002 Plovdiv, Bulgaria; 4 Department of Pharmacognosy and Pharmaceutical Chemistry, Faculty of Pharmacy, Medical University of Plovdiv, 4002 Plovdiv, Bulgaria; paolina.lukova@mu-plovdiv.bg; 5 Department of General and Clinical Pathology, Faculty of Medicine, Medical University of Plovdiv, 4000 Plovdiv, Bulgaria; iliya.bivolarski@mu-plovdiv.bg (I.B.); mariya.koleva@mu-plovdiv.bg (M.K.); 6 Institut Pascal, Clermont Auvergne INP, Université Clermont Auvergne, CNRS, 63000 Clermont-Ferrand, France; cedric.delattre@uca.fr; 7 Institut Universitaire de France (IUF), 1 Rue Descartes, 75005 Paris, France

**Keywords:** transferosomes, sodium humate, dermal patch, wound-healing

## Abstract

**Background**/**Objectives:** For successful wound management, dressings must be maintained in a moist environment to optimally enhance the microenvironment of the wound and efficiently deliver bioactive agents. Sodium humate has demonstrated potential wound-healing activity, although its topical delivery is still a challenge. This study aimed to develop and optimize polysaccharide-based dermal patches incorporating sodium-humate-loaded transferosomes and to assess their physicochemical and wound-healing properties. **Methods:** Transferosomes were obtained via thin-film hydration and prepared utilizing the Taguchi experimental design based on the impact of lipid content, lipid-to-surfactant ratio, and lipid-to-drug ratio on vesicle size, ζ-potential, and drug entrapment efficiency. The optimized transferosomes were loaded into alginate/HPMC composite dermal patches prepared through solvent evaporation. **Results:** The optimized transferosome formulation had an average size of 250.9 ± 2.3 nm, a ζ-potential of −3.57 ± 0.25, a high deformability of 93.01 ± 2.41%, and an effective drug-entrapment efficiency of 30.13 ± 1.04%. The use of transferosomes greatly affected patch thickness, moisture content, and surface morphology. A biphasic drug release profile of sodium humate was demonstrated via an in vitro release study, showing an initial burst followed by sustained drug release within 6 h. In vivo evaluation of transferosome-loaded patches showed that the formulations were able to effectively promote wound healing compared with the control. **Conclusions:** The developed transferosome-embedded alginate/HPMC dermal patches constitute a promising platform for the controlled topical administration of sodium humate and show promising enhancement of wound healing.

## 1. Introduction

Wound healing involves overlapping phases (hemostasis, inflammation, tissue proliferation, and remodeling), which rely on well-regulated cell migration, extracellular matrix production, angiogenesis, and immune responses [1,2]. Acute and chronic wound treatment remains a clinical challenge despite advances in wound care and biomaterials, especially when prolonged inflammation, high oxidative stress, microbial infection, or impaired tissue regeneration interfere with normal wound-healing processes. These often result in delayed wound closure, an enhanced risk of infection, and abnormal scarring, which in turn, lengthens the skin recovery [3]. Chronic wounds are a significant burden on the healthcare system due to their high incidence, tendency to recur, and resistance to standard treatments [4].

Consequently, there is a demand for advanced wound-care practices aimed at altering the wound microenvironment, inhibiting inflammation, guarding against infection, and aiding in tissue repair and regeneration [5]. Bioactive substances derived from natural sources have attracted increased interest in the field of wound healing in recent years. Such interest is also motivated by their inherent biocompatibility, diverse structures, and a wide range of biological activities. Natural compounds are mainly extracted from plants, microbes, or minerals and are preferred over synthetic ones because they are less cytotoxic, better tolerated, and less likely to induce a negative immune or inflammatory response [6]. A wide variety of natural substances also exhibit multifunctional attributes, serving as antioxidants, anti-inflammatory agents, antimicrobial agents, and regenerative agents, which enable the treatment of various pathological factors limiting wound-healing capacity [7].

Among naturally occurring substances, humic acid (and its sodium salt, sodium humate) has emerged as a promising candidate for wound-care applications [8,9,10]. It is a complex organic substance produced by the degradation and transformation of plant detritus and microbial biomass through environmental processes over time. Humic acid is an important component of humic substances found in soil, peat, lignite, and aquatic environments [10]. It is heterogeneous and amorphous, containing various functional groups, including carboxyl, phenolic, hydroxyl, and quinone units. The diversity in the structure leads to higher chemical reactivity and greater interaction with biological membranes, metal ions, and reactive oxygen species, leading to a wide range of biofunctional properties [11]. As a natural bioactive substance, humic acid is becoming a powerful therapeutic tool in both dermatology and wound-care research. Due to its remarkable antioxidant, anti-inflammatory, and antimicrobial properties, it has drawn considerable interest. These features imply therapeutic potential for tissue restoration and healing, modulation of inflammation, and protection of damaged skin [12]. Yet, while these bioactivities are quite promising, the use of humic acid in topical or transdermal formulations is challenging due to its large molecular weight and complex structure. Additionally, its high intrinsic hydrophilicity restricts its penetration through the stratum corneum—the main barrier that controls skin absorption [13,14]. Therefore, topical application is usually associated with limited drug penetration to reach therapeutic concentrations at deeper skin layers, which restricts its clinical applicability in wound healing.

In order to overcome these limitations, advanced delivery systems that improve dermal transport and provide sustained, localized delivery of drugs are necessary. Vesicular nanocarriers, specifically transferosomes, have been proposed as a new option to optimally enhance the topical and transdermal delivery of bioactive compounds. Transferosomes, small, ultra-deformable lipid vesicles mainly consisting of phospholipids and edge activators (such as surfactants), are particularly important for their exceptional flexibility [15]. This structural property enables transferosomes to adjust their shape reversibly, allowing them to diffuse through thin intercellular channels in the stratum corneum [16]. Unlike traditional liposomes that are usually limited to superficial skin layers, transferosomes penetrate using the transdermal hydration gradient. This osmotic gradient enables them to penetrate deeper without inflicting irreversible damage to the integrity of the skin barrier. Transferosomes thus facilitate higher drug delivery to the viable epidermis and dermis yet do not compromise skin integrity [17]. Their vesicle structure also provides a barrier to encapsulated bioactive compounds, which increases their stability and reduces the tendency to undergo early degradation following topical application [18].

Topical or dermal delivery systems of humic acid, such as poultices, emulsions, hydrogels, and patches, have been reported in various studies and have potential for skin therapeutic applications [10,19,20,21]. However, these systems tend to have issues such as limited skin penetration and uncontrolled release rates. Although ultradeformable vesicles are suitable for improving the skin transport of hydrophilic and large-molecular-weight substances, no reports so far have discussed the application of humic acid in transferosomal delivery systems. The incorporation of humic acid (sodium humate) in transferosomal carriers could greatly improve its dermal bioavailability and its therapeutic properties. The encapsulation in these ultradeformable vesicles can overcome the physicochemical barriers of humic acid, protect its bioactive groups, and promote sustained, local delivery at the wound area. The application of transferosome-loaded humic acid via an appropriate topical platform, such as a polysaccharide-based patch system, could also offer synergy, with increased skin penetration, longer retention, and controlled release, thereby enhancing wound-healing effects [22,23].

The aim of this study was to develop transferosomes containing sodium humate optimized in terms of size, stability, and entrapment efficacy and to construct polysaccharide-based dermal patches as their carriers to enhance wound healing. Hybrid patch systems were developed to provide controlled delivery, enhanced retention, and effective wound healing by combining the natural biocompatibility and film-forming potential of polysaccharides (sodium alginate and hydroxypropyl methylcellulose) with the penetration capability of transferosomes.

## 2. Materials and Methods

### 2.1. Chemicals and Reagents

Humic acid sodium salt (sodium humate), phosphatidylcholine (from egg yolk), sodium alginate (from brown algae, medium viscosity ≥ 2000 cP, 2% at 25 °C), hydroxypropyl methylcellulose (~86 kDa, viscosity 2600–5600 cP, 2% at 20 °C), and TWEEN^®^ 80 (Polysorbate 80) were purchased from Sigma-Aldrich (St. Louis, MO, USA). All other reagents were of analytical grade. Zoletil and Meloxidyl were purchased from the local distributors of Virbac (Virbac Corp., Carros, France) and ACCORD (Accord Healthcare Limited, Harrow, UK), respectively. One liter of 70% ethyl alcohol (Chemax Pharma, Sofia, Bulgaria) and the standard product Herbal wonder^®^ (Bioherba, Sofia, Bulgaria) were obtained from a local pharmacy. Eosin Y (1% aqueous solution, cat. no. 294/EOY-10-OT-2.5L), formaldehyde 4% (10% neutral buffer, cat. № 294/FNB4-10L), Histanol 100 (cat. No 294/H100-5L), Histanol 95 (cat. no. 294/H95-5L), hematoxylin G3 (cat. no. 294/HEMG3-OT-2.5L), acetone (cat. no. 48/3413/5), and xylol (cat. no. 348/3410/20) were purchased from BIOCARE Medical (Pacheco, CA, USA). Flexible cohesive bandages (“b-flex”, 4.5 cm × 5 cm) were purchased from the importer, Toshev Farma Ltd. (60 Panayot Volov str., Shumen, Bulgaria).

### 2.2. Preparation of Transferosomes

**Figure 1:**
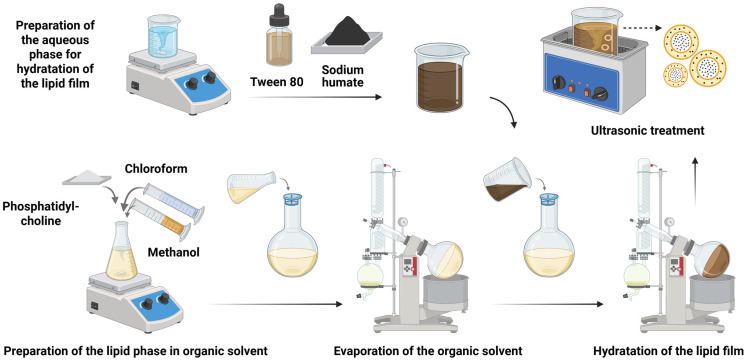
Schematic illustration of the thin-layer hydration method used for the preparation of humic-acid-loaded transferosomes (Created in Biorender. P. Katsarov. (2026) https://BioRender.com/9pluofp).

Transferosomes were prepared using a modified lipid-thin-film hydration method [24], as shown in Figure 1. A solution of phosphatidylcholine (lipid phase) in chloroform and methanol (2:1, *v*/*v*) was prepared and transferred to a dry round-bottom flask. The lipid phase concentration used for transferosome preparation was varied at 5.0, 7.5, and 10.0 mM. The organic solvent was then evaporated using the BUCHI RII Rotavaport rotary vacuum evaporator (BÜCHI Labortechnik AG, Flawil, Switzerland) under reduced pressure and at a temperature of 40 °C. Rotation was started at 20 rpm and gradually increased to 200 rpm to obtain a uniform thin lipid film along the inner flask surface. The formed film was kept under vacuum for 2 h to completely evaporate the solvent. The lipid film was hydrated with an aqueous solution of the surfactant (Tween 80) and sodium humate while being rotated continuously at 100 rpm for 30 min at room temperature and was then allowed to equilibrate and swell for 2 h. Phosphatidylcholine-to-surfactant ratio (9:1, 17:3, 4:1) and phosphatidylcholine-to-sodium humate ratio (5:1, 10:1, 15:1) were varied for the different transferosome models, with the robust statistical design of the L9_3 Taguchi experiment being applied [25]. The resulting liposome structures were subjected to ultrasonic treatment for 30 min using a Sonorex SUPER RK sonicator (Bandelin electronic, Berlin, Germany) to optimize their size. After sonication, the transferosome suspension was stored at 4 °C to allow complete vesicle formation. All transferosome preparations were performed in triplicate to ensure reproducibility. The data obtained from the experimental design were statistically analyzed using Minitab^®^ Statistical Software version 21.1 (Minitab LLC, State College, PA, USA; 2023).

### 2.3. Characterization of Transferosomes

#### 2.3.1. Particle Average Size, Particle Size Distribution, and ζ-Potential

After the non-encapsulated sodium humate was removed, the formulated transferosomes with sodium humate were characterized in terms of average size, particle size distribution, and ζ-potential using dynamic and electrophoretic light scattering with a particle size analyzer (Zetasizer UltraRed, Malvern Panalytical Ltd., Malvern, UK). All measurements were performed within 48 h of model preparation and conducted in triplicate.

#### 2.3.2. Drug-Entrapment Efficiency (DEE)

The entrapment efficiency of sodium humate in the formulated transferosomes was determined by an indirect method, involving centrifugation of the liposome suspension to remove the unentrapped active substance. The transferosomes solution was centrifuged at 18,000 rpm for 30 min using a Sigma 3-18KS centrifuge equipped with a fixed-angle rotor (Sigma Laborzentrifugen GmbH, Osterode am Harz, Germany) to separate the vesicles from any unentrapped drug. The supernatant, containing free sodium humate, was analyzed using a UV-Vis Evolution 300 spectrophotometer (Thermo Fisher Scientific, Waltham, MA, USA). The quantitative determination of sodium humate was carried out using the absorbance at a wavelength of 254 nm, which is characteristic of the drug substance [26,27]. Drug-entrapment efficiency (DEE) (%) was calculated as the ratio of the indirectly determined drug concentration in the vesicles (the difference between the total drug concentration and the total free drug concentration) to the total drug concentration [22]. All experiments were performed in triplicate, and the mean values were reported.

#### 2.3.3. Transmission Electron Microscopy (TEM)

The formulated humate-loaded transferosomes were analyzed by transmission electron microscopy (TEM) (Talos F200C Thermo Fisher Scientific, Waltham, MA, USA). Suspensions of the nanovesicles were drop-cast on 200-mesh formvar/carbon-coated copper grids and imaged at 200 kV.

#### 2.3.4. Extrusion-Based Deformability Assessment

The comparative deformability of the obtained vesicles was determined via extrusion. Ten milliliters of transferosome suspension were pushed through a 200 nm polycarbonate membrane using a syringe under constant manual pressure for 10 min. Vesicle sizes were measured before and after extrusion with a Zetasizer UltraRed particle size analyzer (Malvern Panalytical Ltd., Great Malvern, UK). Deformability (%) was calculated on the basis of the following equation [22,28,29]:

(1) $$Deformability\;(\%)=\frac{Vesicle\;size\;after\;extrusion}{Vesicle\;size\;before\;extrusion}\times 100$$

### 2.4. Formulation of Dermal Patches

**Figure 2:**
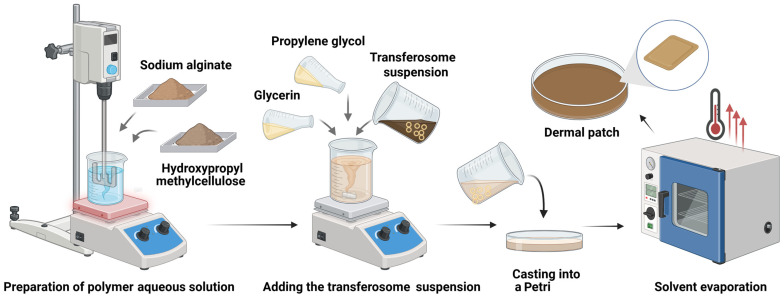
Schematic illustration of the solvent evaporation method used for the preparation of single-layer dermal patches (Created in Biorender. P. Katsarov. (2026) https://BioRender.com/9s3pjj9).

Polysaccharide-based dermal patches were prepared using the solvent evaporation method through the addition of glycerin, propylene glycol, and transferosomes loaded with sodium humate to an aqueous solution of sodium alginate and hydroxypropyl methylcellulose (HPMC) (Figure 2).

To outline an optimal patch formulation, the ratio between the two polysaccharides was varied (1:1, 1:2, and 2:1) at a constant polymer concentration of 5% *w*/*v*, a plasticizer concentration of 30% *v*/*v* (glycerin and propylene glycol at 1:2 ratio), and drug-loaded transferosome equivalent of 140 mg sodium humate (dermal patches with high concentration of sodium humate) or 70 mg sodium humate (patches with low concentration of sodium humate). The polymer solution was prepared by dissolving sodium alginate and HPMC in purified water under continuous stirring at 500 rpm for 2 h using a magnetic stirrer at 60 °C. Glycerin and propylene glycol, as plasticizers, and transferosome were gradually added to the solution while being stirred, and the mixture was maintained at room temperature for 30 min. The mixture was then cast into a Petri dish (150 mm diameter, 20 mm height) left to dry in a dryer (oven) at 40 °C for 12 h and then stored in a desiccator. After complete drying, the formulated single-layer patches were cut into pieces with an area of 8 cm^2^. Two dermal patch (DP) models were therefore designed (low and high drug concentration, LC and HC) with humic acid concentrations of 0.4 mg/cm^2^ (DP-LC) and 0.8 mg/cm^2^ (DP-HC), respectively, and patches without drug (a transferosome-free model) were also formulated for control (DP-Contr). Each formulation was prepared in triplicate.

### 2.5. Characterization of Dermal Patches

#### 2.5.1. Scanning Electron Microscopy (SEM)

The formulated dermal patches were analyzed for thickness and surface morphology using the Prisma E SEM (Thermo Scientific, Waltham, MA, USA). The dry samples were coated with gold using a Q150T ES Plus sputter coater (Quorum technologies, East Sussex, UK), and then micrographic images at appropriate magnification were recorded at 15 kV acceleration voltage.

#### 2.5.2. Moisture Content

The moisture content of dermal patches was determined using a moisture analyzer Kern MLB (KERN & Sohn GmbH, Balingen, Germany) based on the thermogravimetric principle. Dermal patches, cut into uniform pieces (4 cm × 2 cm), were placed on the sample pan of the moisture analyzer and were accurately weighed. The samples were heated at 105 °C until a constant weight was reached. Moisture content was calculated automatically and expressed as the percentage weight loss relative to the initial sample weight [30]. Each measurement was performed in triplicate, and results are reported as the mean value ± standard deviation.

#### 2.5.3. In Vitro Drug Release

The in vitro dissolution study was conducted using the stirred beaker method. Patch samples (4 cm × 2 cm) were placed into a beaker containing 20 mL pf PBS (phosphate buffer saline) with a pH of 6.8 at 37 ± 0.5 °C, and the rate of stirring was 50 rpm. Samples were taken at set time intervals and replaced with an equivalent volume of fresh medium. All the samples were filtered (0.22 μm PTFE syringe filters, Isolab Laborgeräte GmbH, Eschau, Germany) and analyzed with UV-VIS spectrophotometry (Section 2.3.2). Triplicate average values with standard deviation are reported.

### 2.6. In Vivo Wound-Healing Analysis

#### 2.6.1. Animals and Treatment

Male Wistar rats (body weight 200–250 g) were used for the experiments. The animals were housed under standard laboratory conditions: 22 ± 1 °C, 45% relative humidity, and a 12 h light/12 h dark cycle, with unrestricted access to standard laboratory chow and water. They were randomly assigned to four groups, each group consisted of eight animals, and they were housed for 7 days for acclimatization. After the infliction of wounds, each animal was housed in an individual polyethylene cage (L × W × H:48 × 35 × 20 cm).

We followed a protocol for wound infliction and treatment described in literature [31]. Briefly, on the first day of the experiment, the animals were anesthetized by intramuscular injection of 80 mg/kg of Zoletil^®^ (tiletamine HCL + zolazepam). The hair on the back of the rats was shaved, and 70% ethyl alcohol was applied to the skin with a cotton pad to prevent infection. In the posterior dorsal region of each rat, two wounds were inflicted using a punch with a diameter of 8.0 mm. They were disinfected using 70% ethyl alcohol and photographed with a Nikon D3400 digital camera (Nikon Corp, Tokyo, Japan) at 55× magnification, with the camera being 26 cm distant from the wound. The wounds of each animal were treated according to the respective group. The following groups were evaluated:

- Group 1: control—treated with DP-Contr;
- Group 2: reference treatment—a standard product for wound healing (Herbal wonder^®^);
- Group 3:dermal patch DP-LC;
- Group 4: dermal patch DP-HC.

The two wounds of each animal from groups 1, 3, and 4 were covered with the respective patch, and a cotton gauze was placed over the patch. The patch and the gauze were fixed to the rat’s body with a self-adhering, elastic, and water-resistant bandage (“b-flex”, 4.5 cm × 5 m). The rats from group 2 were treated with Herbal wonder^®^ ointment, which was applied to the wound using a sterile cotton pad. The wounds were covered with cotton gauze and vet wraps. A single oral dose of 2 mg/kg of Meloxidyl^®^ (meloxicam) was administered via gastric tube to each animal to prevent postoperative pain and discomfort. The animals were placed in individual cages and observed for the next 24 h. Animals showing signs of pain or discomfort were given an additional dose of 1 mg/kg of Meloxidyl^®^.

The patches or the reference product were removed and reapplied every day, once in the morning, for a time period of 11 days. On days 3, 5, 7, 9, and 11, photographs were taken as described above before application of the respective treatment. The rats were euthanized on day 11 of the experiment by fracture of the spine in the neck area after administration of Xylazine 5 mg/kg b.w., and the whole two wounds of each animal with 5 mm margins were cut out and subjected to histopathological examination.

#### 2.6.2. Determination of Wound-Healing Percentage

We employed a study protocol described in literature [32]. The macroscopic evaluation of the wound-healing process was performed using digital photographs of the wounds taken on days 1, 3, 5, 7, 9, and 11. To measure the wound area, image analysis software (ImageJ 1.52a, NIH) was used. The percentage of wound healing was calculated by the following formula:

(2) $$Wound\;healing\;(\%)=\left(\frac{A1-An}{A1}\right)\times 100$$

where *A*1 is the wound area on day 1 (the day of the surgery), and *An* is the wound area on the specific day.

#### 2.6.3. Histopathological Observation

The experimental protocol was based on the description of other authors [33]. The excised wounds were fixed in a 10% neutral-buffered formalin solution and embedded in paraffin blocks. The process of histological sample preparation was described previously [34]. The paraffin blocks were cut into thin slices (5 μm thickness), followed by hematoxylin and eosin staining. The samples were observed with Leica DM500 (Leica Microsystems, Deerfield, IL, USA) and Zeiss AXIO Scope A1 microscopes (Carl Zeiss Microscopy GmbH, Jena, Germany) independently by two pathologists blinded to the experimental groups. Each sample was scored according to the following scale [33]:

- Score 1–3 indicates absent or only minimal fibroblast accumulation, with no evidence of granulation tissue formation or epithelial migration.
- Score 4–6 corresponds to a thin, early granulation matrix primarily composed of inflammatory cells, accompanied by sparse fibroblasts, limited capillary presence or collagen deposition, and only minimal epithelial migration.
- Score 7–9 reflects a moderately developed granulation tissue characterized by abundant inflammatory cells, increased fibroblast numbers, greater collagen deposition, pronounced neovascularization, and minimal to moderate epithelial migration.
- Score 10–12 represents well-formed, highly vascular, granulation tissue rich in fibroblasts with substantial collagen deposition, along with epithelial coverage extending from partial to complete closure of the wound surface.

### 2.7. Statistical Analysis

Statistical analysis of the transferosome data was conducted using Minitab^®^ Statistical Software version 21.1 (Minitab LLC, State College, PA, USA; 2023) (Section 2.2). Statistical analysis of the data obtained in the in vivo experiments was performed with SPSS software version 17.0 (IBM, Armonk, NY, USA). Results are presented as the mean ± standard error of mean. Differences between group means were evaluated by one-way analysis of variance (ANOVA) and Tukey’s post hoc test. Statistical significance was reported when *p* ≤ 0.05.

## 3. Results and Discussion

### 3.1. Optimization of the Transferosome Formulation Process

The initial objective of this study was to optimize the formulation of transferosomes in order to obtain vesicles with physicochemical characteristics suitable for transdermal drug delivery, namely an appropriate nanoscale size, acceptable ζ-potential indicative of colloidal stability, and high drug entrapment efficiency. A Taguchi L9 experimental design was used to evaluate the impact of the most important technological parameters on the characteristics of the transferosome. The selected independent variables were lipid concentration (mM), lipid–surfactant ratio (M/M), and lipid–sodium humate (HaNa) ratio (*w*/*w*), each studied at three predefined levels. These factors were selected based on their significant impact on vesicle formation, membrane flexibility, and efficiency of drug incorporation. The experimental design matrix, along with the corresponding factor levels and the nine formulated transferosome models, is presented in Table 1. The prepared formulations were evaluated in terms of average vesicle size (nm), ζ-potential (mV), and entrapment efficiency (%), which were selected as the primary dependent responses due to their relevance to transdermal performance and formulation stability.

The experimental results were analyzed using the Taguchi methodology to determine the relative contributions and influences of each formulation parameter on the studied responses. Also, regression analysis was performed to clarify the quantitative relationships between the independent variables and the dependent outcomes, enabling identification of the most influential variables and prediction of optimal formulation conditions. The results of Taguchi analysis and regression modeling with respect to their impact on vesicle size, surface charge, and drug-entrapment efficiency are discussed in the subsections below (Table 2).

#### 3.1.1. Influence of the Varied Parameters on the Average Transferosome Size

Vesicle size is an important factor in the transdermal drug delivery of transferosomal systems, as it affects skin penetration, drug release, and formulation stability. Ultradeformable vesicles, typically an average 100–300 nm in size, can pass through the skin’s hydration-gradient-induced narrow pathways, while larger vesicles tend to remain on the skin surface [35]. Accordingly, optimizing and preserving vesicle size is critical for efficient transdermal delivery and consistent therapeutic outcomes.

The conducted Taguchi analysis revealed that lipid concentration had the most significant impact on the average transferosomes size. Statistical data are presented in Table 2.

As the lipid concentration increased, the signal-to-noise ratio dropped by 6.73 points, resulting in the highest delta value, which was also confirmed by the high positive coefficient (71.79) obtained from the regression analysis. This indicated that when the lipid concentration was increased, it significantly increased the average transferosome size. This can be explained with Smoluchowski’s coagulation theory, which states that the rate of aggregation is proportional to the square of the particles’ concentration. A similar phenomenon was also confirmed by other authors [36]. The data gained from the analysis of variance confirmed that lipid concentration is the most dominant, statistically significant factor, showing an F-value of 56.55 and a *p*-value of 0.001. A higher F-value indicates that changes in the examined factor produce a stronger effect on the measured response, while lower *p*-values indicate a higher level of statistical significance. Therefore the lowest varied lipid concentration of 5 mM was outlined as optimal, resulting in vesicles with the smallest average size.

The lipid–surfactant ratio also had a significant effect on the average transferosome size, showing a delta value of 3.10. The vesicle average size decreased with the decrease of the lipid–surfactant ratio (or an increase in surfactant concentration). This was probably due to the steric stabilization provided by Tween 80 to the vesicular structure [37]. The subsequent analysis of variance confirmed the statistical significance of lipid–surfactant ratio, which showed an F-value of 12.24 and a *p*-value of 0.017.

The third varied parameter, lipid–Na-humate ratio, showed a delta value close to zero (0.57), indicating that the impact of sodium humate within the range of variation was negligible. This was also confirmed by the analysis of variance, showing an F-value of 0.69 and a *p*-value of 0.445, which indicated it to be statistically insignificant.

Overall, the data showed that the applied model was statistically reliable in explaining the observed variation, showing an R^2^-value of 93.29%. The R^2^-adj value (89.26%) was close to the value of R^2^, which signified the good correlation of the model. The high F-value (23.16) and *p*-value of 0.002 from the regression analysis indicated that the relationship observed by this model was not due to random chance.

#### 3.1.2. Influence of the Varied Parameters on the Transferosomes’ ζ-Potential

Regarding the ζ-potential, the lipid–surfactant ratio was the most dominant factor, showing a very high delta value of 31.815 and a change in the ζ-potential from a negative value to almost zero as the lipid–surfactant ratio decreased (i.e., the concentration of Tween 80 increased). This behavior can be explained by the displacement of the shear plane theory and the principles of instrumental ζ-potential measurement [38,39]. The analysis of variance showed that this factor had a statistically significant impact on the ζ-potential, with an F-value of 74.07 and a *p*-value of <0.001 (Table 2). However, despite the negative effect on the ζ-potential, the stability of the vesicles should not be compromised because polysorbates (Tween 80) provide steric stabilization to the vesicles [40].

The second most dominant factor regarding the ζ-potential was the lipid–sodium humate ratio, with a delta value of 8.947 and a clear trend of increasing ζ-potential as the ratio decreased (i.e., as the concentration of sodium humate increased). This was likely due to the anionic nature of sodium humate, which could lead to a slight increase in the negative range of the ζ-potential [41]. However, the impact of the lipid–sodium humate ratio was determined to be statistically insignificant, with a relatively small F-value (1.51) and a *p*-value of 0.274. The lipid concentration was determined to have the least impact on the ζ-potential (delta-value 4.834) and had an F-value of 0.01, indicating that the lipid concentration has practically no effect on the ζ-potential, as expected because electroneutral phospholipids were used for the formation of the transferosomes.

#### 3.1.3. Influence of the Varied Parameters on the Transferosome Drug-Entrapment Efficiency

The lipid–sodium humate ratio had the most significant impact on drug-entrapment efficiency, with a delta value of 6.04, three times higher than the second most significant factor, lipid concentration, which had a delta value of 1.83. There was a strong negative correlation with the lipid–sodium humate ratio, as confirmed by the regression analysis coefficient of −10.753. As the ratio increased (with the increase in the sodium humate concentration), entrapment efficiency decreased significantly. Because sodium humate is a hydrophilic molecule, this behavior can be explained by the geometric saturation of the transferosomes, which decrease entrapment efficiency [42]. The analysis of variance showed that the factor had a statistically significant impact on entrapment efficiency, with a very high F-value of 159.17 and a *p*-value of <0.001 (Table 2).

The lipid concentration showed a delta value of 1.83. There was a tendency for entrapment efficiency to increase with increasing lipid concentration, which could be due to an increase in the total volume of “internal water” available to entrap the drug [43]. This was also confirmed by the regression analysis, which showed a positive coefficient (1.217). The analysis also showed that the factor had a statistically significant influence on entrapment efficiency (*p*-value = 0.016). The variable with the smallest effect on entrapment efficiency was the lipid–surfactant ratio (delta value of 1.2). The influence of the entrapment efficiency was statistically insignificant, showing a *p*-value > 0.05.

### 3.2. Characterization of the Optimal Model Transferosomes

**Figure 3:**
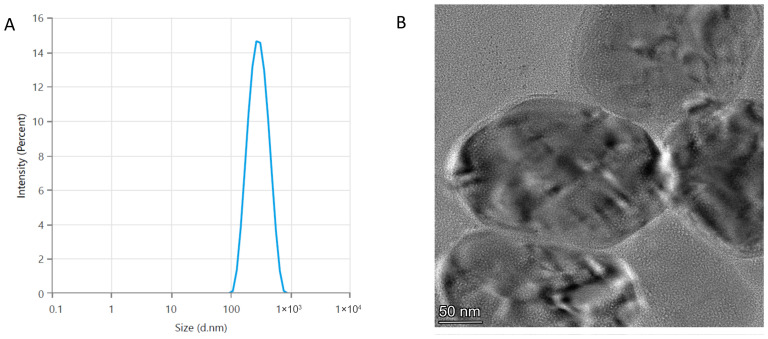
Characterization of sodium-humate-loaded transferosomes from the optimal model TS.1.2.2: (**A**) particle size distribution and (**B**) TEM image.

Among the formulations examined, the model TS.1.2.2 (prepared at a lipid concentration of 5 mM, a lipid–surfactant ratio of 17:3, and a lipid–sodium humate ratio of 10:1) had the smallest average vesicle size (250.9 nm) and relatively high drug-entrapment efficiency (30.13%). Due to these characteristics, model TS.1.2.2 was outlined as suitable for inclusion in dermal patch formulations for transdermal and wound-healing applications. Figure 3 shows the particle size distribution and morphological characteristics of the optimized transferosome model determined with dynamic light scattering and transmission electron microscopy, respectively. There was a monomodal particle size distribution, which indicated a relatively uniform vesicle population with minimal aggregation and a relatively narrow size distribution.

The optimized transferosome model TS.1.2.2 showed a deformability value of 93.01% ± 2.41. These results indicated that the designed vesicles were ultradeformable and could enter pores smaller than their original size while retaining their structural integrity. Such high deformability is a key characteristic of transferosomes, and it is considered essential for effective skin penetration, as it enables vesicles to traverse narrow intercellular pathways of the stratum corneum under physiological pressure gradients without vesicle rupture.

### 3.3. Formulation and Characterization of Dermal Patches

The next step of this work was to incorporate the optimized sodium-humate-loaded transferosomes into polysaccharide-based dermal patches in order to develop a composite system suitable for wound-healing applications. A preliminary formulation study was performed to optimize the ratio between sodium alginate and HPMC in order to select the most suitable polymeric matrix for dermal patches.

Polysaccharide-based patches were prepared by varying the sodium alginate–HPMC ratio (2:1, 1:1, and 1:2), while polymer concentration, plasticizer content, and drug-loaded transferosome amounts were kept constant. Preliminary formulation considerations suggested that increasing the glycerin fraction leads to highly hydrated and overly soft films, whereas propylene-glycol-dominated systems improve the mechanical integrity of the patch. A glycerin–propylene glycol ratio of 1:2 was selected as optimal, providing sufficient flexibility while limiting excessive moisture uptake and preserving the structural stability of the alginate/HPMC matrix.

The results showed that the sodium alginate–HPMC ratio strongly influenced the thickness and moisture content of the patches. Patches with higher alginate content (2:1) were thicker, with higher residual moisture and a softer texture, whereas those with higher HPMC content (1:2) were thinner, with lower moisture and better elasticity. These differences can be explained by the distinct physicochemical properties of the two polysaccharides. Sodium alginate is a highly hydrophilic, anionic polymer with a strong affinity for water due to carboxylate groups along its backbone. This results in substantial bound moisture even after drying of alginate-rich matrices and the formation of thicker, softer films that exhibit more hydrogel-like behavior. Moreover, increased hydration limits polymer chain packing during solvent evaporation, thereby increasing film thickness [44].

HPMC, on the other hand, is a well-known film-forming polymer that facilitates effective chain entanglement and dense matrix formation during drying [45]. When combined with plasticizers such as glycerin and propylene glycol, thinner films are formed, which enhance flexibility by weakening intermolecular interactions. The reduced moisture content of such HPMC-rich patches can be attributed to HPMC’s lower hygroscopicity compared with alginate [46]. Wound-healing dermal patches should remain moist and regenerative for the patient while retaining mechanical performance, elasticity, and ease of handling. Excessive moisture and softness exhibited by alginate-containing formulations can disrupt structural stability and handling, particularly under high-hydration conditions. On the other hand, very thin films with low moisture content, resulting from high HPMC content, may not provide adequate hydration or handle exudates well, thereby hindering healing [47,48]. Therefore, a 1:1 sodium alginate–HPMC formulation was chosen as most suitable for the dermal wound-healing patches. This combination provides good moisture retention, allowing a moist healing environment to persist while providing enough elasticity to stabilize the patch and be comfortable for the patient.

Based on the outlined optimal polysaccharide ratio, two dermal patch models incorporating sodium-humate-loaded transferosomes at different drug concentrations were formulated, and a drug-free patch model was used as a control. The composition of the formulated patch models is presented in Table 3.

The addition of transferosomes to the polysaccharide matrix increased patch thickness (80.25–74.60 µm) and moisture retention (13.23–10.96%) compared with transferosome-free patches (70.25 µm; 8.87%). The effect was greater at higher transferosome concentrations, suggesting disruption of polymer packing and enhanced water retention from the hydrated vesicular structures. In the absence of transferosomes (model DP-Contr), the polymer matrix could pack and densify efficiently during solvent evaporation. Without dispersed nanostructures to disrupt polymer–polymer interactions, thinner films with lower residual water resulted. At low transferosome loading (model DP-LC), vesicles were likely well dispersed within the polymer matrix and acted as mild physical spacers. Their lipid bilayers and associated hydration shells can increase water retention and slightly reduce polymer chain packing efficiency during drying [49,50]. This can lead to modest increases in thickness and moisture content while maintaining good film integrity.

**Figure 4:**
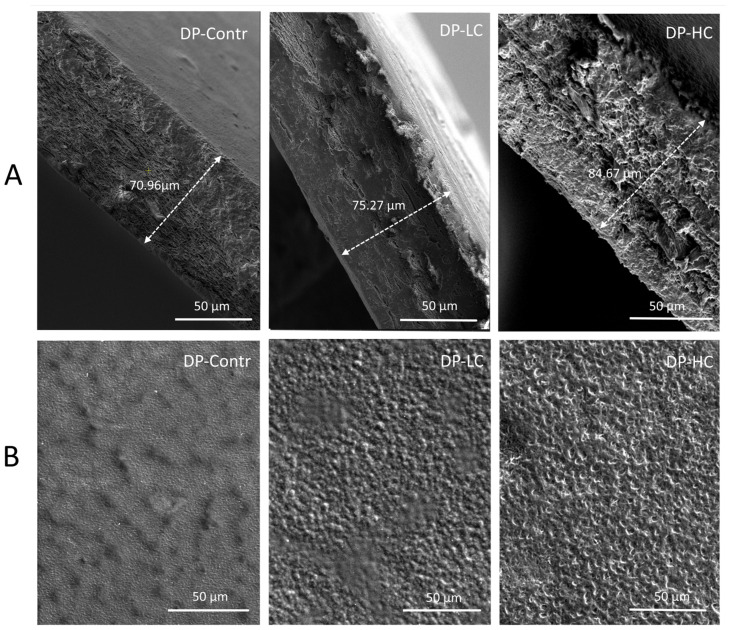
SEM micrographs of (**A**) cross-section (thickness) and (**B**) surface (morphology) of dermal patches without transferosomes (DP-Contr), with low transferosome concentration (DP-LC) and with high transferosome concentration (DP-HC), obtained at 2000× magnification.

The incorporation of transferosomes also influenced the surface morphology of the obtained dermal patches. SEM images of the formulated patch models are presented in Figure 4.

The transferosome-free patches (DP-Contr) exhibited a smoother, more homogeneous surface, consistent with well-packed polysaccharide matrices. Transferosome-containing patches (DP-LC and DP-HC) displayed a more heterogeneous surface morphology, with distinctive irregularities and surface folds that became more prominent with increasing transferosome concentration. These morphological differences can be attributed to dispersed vesicular architectures within the polymeric matrix, which inhibit the uniform packing of polymer chains during film preparation and drying. Other authors reported similar observations, noting that embedding liposomes in alginate-based films disrupted the film’s microstructure, producing surface folds and depressions, as well as vesicle-like structures in the cross-section. These findings were linked to liposomes acting as structural discontinuities or spacers within the polymer network, thereby increasing microstructural heterogeneity [51]. The observed increase in surface heterogeneity in transferosome-loaded patches is consistent with their increased thickness and moisture retention and may be advantageous for wound-healing applications, as a rougher, more hydrated surface can enhance fluid interaction and potentially improve patch–wound contact.

**Figure 5:**
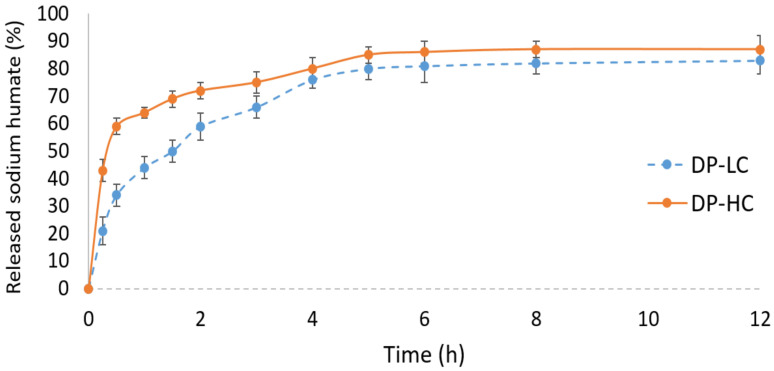
In vitro release profile of sodium humate from dermal patch models DP-LC and DP-HC (*n* = 3).

The sodium humate in vitro release profiles from dermal patches with low (DP-LC) and high (DP-HC) transferosome loadings are shown in Figure 5. Both patches exhibited a biphasic release pattern, with a burst release occurring within the first 60 min, followed by sustained release.

The observed burst release is typical of polymeric patches incorporating vesicular drug carriers and can be associated with the polymer matrix and transferosome bilayers and the rapid diffusion of sodium humate located near the patch surface [52].

The enhanced burst drug release of DP-HC was probably due to higher transferosome (drug) loading, leading to a greater fraction of drug positioned at or near the surface of the patches and enhanced matrix hydration, thereby facilitating faster initial diffusion. In DP-LC, the relatively low transferosome amount likely contributed to better drug distribution within the polymeric network and minimized matrix disturbances, thereby reducing rapid drug release and allowing the initial release to be more controlled.

The subsequent sustained-release phase for both formulations was governed by the diffusion of sodium humate from the hydrated polymer matrix and by gradual release from the transferosome bilayers, with matrix swelling and polymer relaxation contributing to prolonged drug release [53].

### 3.4. In Vivo Evaluation of the Dermal Patches

The in vivo wound-healing efficacy of the developed dermal patch formulations was evaluated in rats with experimentally induced dorsal skin wounds. Wound healing was assessed by macroscopic analysis of wound contraction over an 11-day treatment period. In addition, histopathological examination of excised wound tissues was performed on day 11 to evaluate granulation tissue formation, epithelialization, and overall tissue repair among the experimental groups.

**Figure 6:**
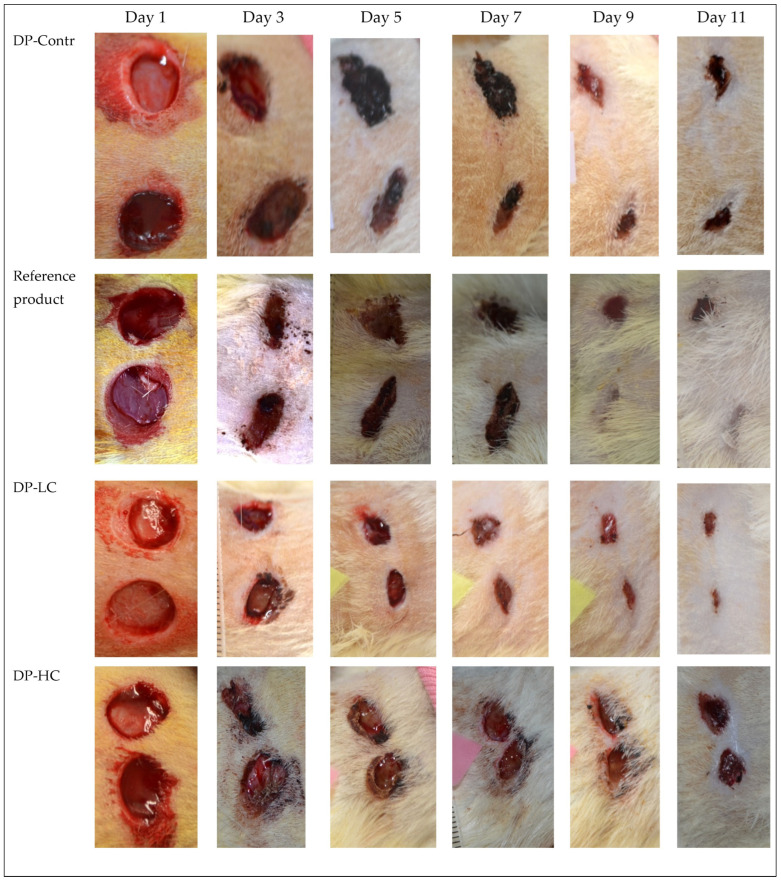
Macroscopic evaluation of wound contraction during treatment with dermal patches without transferosomes (DP-Contr), the reference product (Herbal wonder^®^), dermal patches with low transferosome concentration (DP-LC), and dermal patches with high transferosome concentration (DP-HC).

Macroscopic wound contraction was monitored during the course of treatment on days 1, 3, 5, 7, 9, and 11 (Figure 6).

**Figure 7:**
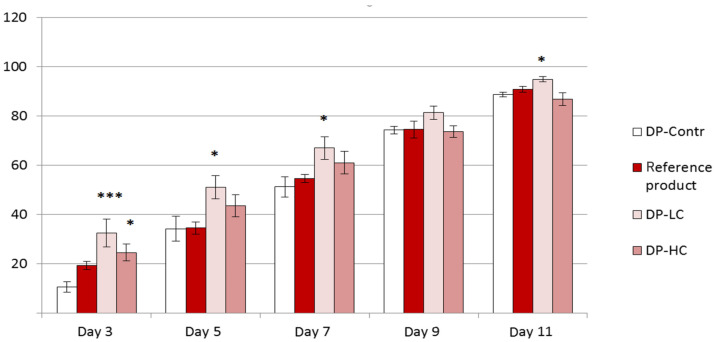
Changes in the percentage of wound healing after treatment with dermal patches. Data are presented as mean ± SEM. The symbol * indicates *p* ≤ 0.05 vs. the control group on the same day; *** indicates *p* ≤ 0.001 vs. the control group on the same day.

On the 3rd day of the experiment, a significant increase in the percentage of wound healing was observed in groups treated with 0.42% and 0.84% sodium humate when compared to the control group (32.54 ± 5.64 vs. 10.51 ± 2.14, *p* < 0.001; 24.48 ± 3.43 vs. 10.51 ± 2.14, *p* < 0.05), as shown in Figure 7. Increased epithelization of the wounds was observed also on days 5, 7, and 11 in the group treated with the formulation containing 0.42% sodium humate compared to the control group on the same day (51.09 ± 4.77 vs. 34.20 ± 5.00, *p* < 0.05 on day 5; 67.00 ± 4.53 vs. 51.25 ± 4.15, *p* < 0.05 on day 7; 94.92 ± 1.05 vs. 88.77 ± 0.94, *p* < 0.05 on day 11).

Accordingly, histopathological analysis of wound tissue was performed on day 11 to assess tissue repair at the microscopic level. The distribution of histopathological scores is presented in Table 4.

**Figure 8:**
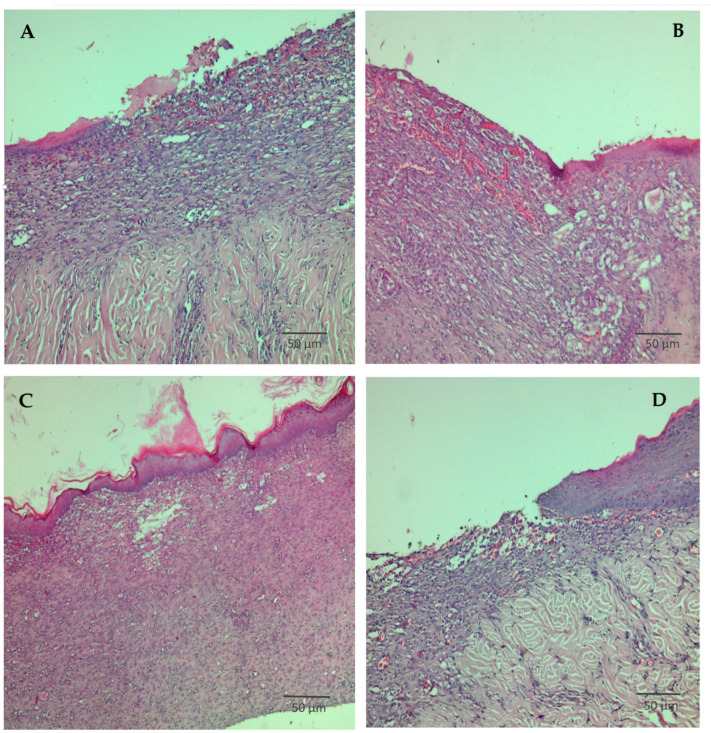
Histological examination of the wound tissue on day 11. Panel (**A**)—treatment with DP Contr.; panel (**B**)—treatment with reference product; panel (**C**)—treatment with DP-LC; panel (**D**)—treatment with DP-HC. Staining: hematoxylin and eosin; Magnification 400×.

Representative histological sections of wound tissue obtained on day 11 are shown in Figure 8. In the group treated with DP-Contr (Figure 8A), the granulation tissue appeared immature, consisting predominantly of a few inflammatory cells accompanied by a small number of fibroblasts, with epithelial migration ranging from minimal to absent.

As shown in Figure 8B, the treatment with the reference product resulted in the formation of moderately thickened granulation tissue, composed mainly of inflammatory cells and fibroblasts, with pronounced neovascularization and a moderate degree of epithelial migration.

In the group treated with DP-LC (Figure 8C), the granulation tissue was markedly thickened, containing a moderate number of fibroblasts and demonstrating complete epithelial coverage.

Figure 8D demonstrates the findings in the group treated with DP-HC: immature granulation tissue, characterized predominantly by isolated inflammatory cells and fibroblasts, with epithelial migration ranging from minimal to absent.

The macroscopic and histological results from experiments conducted on a rat skin wound model show that the sodium humate transferosome-loaded dermal patches accelerate wound healing, with the effect being more pronounced at the lower concentration of sodium humate (DP-LC). This pattern corresponds to a hormetic dose response, namely a biphasic dose/concentration response common in wound healing and marked by stimulation at low doses and inhibition at high doses [54]. While moist environments facilitate healing, excessive moisture and occlusion can produce periwound maceration as well as hinder keratinocyte migration. The use of higher transferosome loading can increase moisture retention and polymer matrix heterogeneity, which may develop an excessively moist/occlusive interface, causing maceration of the surrounding tissue and subsequently limiting healing [55]. Higher vesicle/drug loading has also been associated with enhanced initial burst release and altered sustained delivery, which was confirmed by the data on the sodium humate release profiles presented in Figure 8. The higher initial burst drug release (DP-HC) can result in local irritation or alter the wound biochemistry through strong binding/chelation effects, diminishing late-stage advantages compared with the more balanced sustained drug delivery achieved by the low-concentration patch (DP-LC) [56].

Wound healing is among the body’s most complicated biological processes and occurs through four overlapping stages: hemostasis, inflammation, proliferation, and tissue remodeling [57,58]. It can be assumed that the anti-inflammatory and antioxidant effects of sodium humate described in the literature play a role and contribute to wound healing. Sodium humate enhanced wound healing in the rat model by accelerating wound contraction, increasing hydroxyproline content, and improving overall tissue repair. According to researchers, this pro-healing effect may be mediated through the transforming growth factor-*β* (TGF-*β*)/Smad signaling pathway [9].

## 4. Conclusions

In this study, transferosome-embedded alginate/HPMC dermal patches were successfully developed as a novel platform for the topical delivery of sodium humate for wound-healing applications. Optimization of the transferosome formulation enabled the selection of a vesicular system with suitable size, deformability, ζ-potential, and drug-entrapment efficiency for dermal use. The incorporation of the optimized transferosomes into polysaccharide-based matrices influenced the thickness, hydration behavior, surface morphology, and drug release characteristics of the obtained dermal patches. The performed evaluation in wound models in vivo demonstrated that sodium humate transferosome-loaded patches enhanced wound healing compared to the drug-free controls, confirming the therapeutic potential of the developed system. Overall, the results indicate that alginate/HPMC dermal patches incorporating sodium-humate-loaded transferosomes represent a promising approach for wound management, combining favorable physicochemical properties with effective biological performance. Further studies focusing on long-term stability, detailed mechanical evaluation, and clinical translation may support their future application in wound-care therapy.

## Figures and Tables

**Table 1:** Design matrix of the Taguchi L9 design with formulation variables and corresponding transferosome models (*n* = 3), (SD—standard deviation; PDI—polydispersity index; DEE—drug entrapment efficiency).

Model	Lipid Concentration		Lipid–Surfactant Ratio		Lipid–Na-Humate Ratio		Average Size	PDI	ζ-Potential	DEE
	Level	mM	Level	M/M	Level	*w*/*w*	nm ± SD		mV ± SD	% ± SD
TS.1.1.1	1	5.0	1	9:1	1	15:1	380.6 ± 5.4	0.3655	−9.49 ± 0.13	40.49 ± 2.12
TS.1.2.2	1	5.0	2	17:3	2	10:1	250.9 ± 2.3	0.2079	−3.57 ± 0.25	30.13 ± 1.04
TS.1.3.3	1	5.0	3	4:1	3	5:1	304.5 ± 3.4	0.3887	−0.69 ± 0.05	18.20 ± 1.73
TS.2.1.2	2	7.5	1	9:1	2	10:1	580.6 ± 1.7	0.5467	−12.45 ± 0.83	32.17 ± 3.78
TS.2.2.3	2	7.5	2	17:3	3	5:1	430.5 ± 3.7	0.2597	−5.13 ± 0.33	20.13 ± 2.13
TS.2.3.1	2	7.5	3	4:1	1	15:1	316.7 ± 4.3	0.1471	−0.07 ± 0.01	42.18 ± 4.42
TS.3.1.3	3	10.0	1	9:1	3	5:1	751.7 ± 4.1	0.5063	−10.96 ± 0.64	27.83 ± 5.73
TS.3.2.1	3	10.0	2	17:3	1	15:1	670.6 ± 1.6	0.6367	−2.71 ± 0.32	48.01 ± 2.82
TS.3.3.2	3	10.0	3	4:1	2	10:1	590.6 ± 2.8	0.6109	−0.46 ± 0.11	31.23 ± 4.31

**Table 2:** Response table for signal-to-noise ratios and analysis of variance.

Variable		Lipid Concentration (mM)			Lipid/Surfactant (M/M)			Lipid/Sodium Humate (*w*/*w*)		
Impact On		Average Size *	ζ-Potential **	DEE **	Average Size *	ζ-Potential **	DEE **	Average Size *	ζ-Potential **	DEE **
Signal to-noise ratios levels	1	−49.76	9.136	28.98	−54.80	20.749	30.40	−52.72	1.660	32.76
	2	−52.66	4.302	29.58	−52.40	11.301	29.76	−52.90	8.716	29.87
	3	−56.49	7.546	30.80	−51.70	−11.066	29.20	−53.29	10.607	26.72
	Delta	6.73	4.834	1.83	3.10	31.815	1.20	0.57	8.947	6.04
	Rank	1	3	2	2	1	3	3	2	1
Analysis of variance source	DF	1	1	1	1	1	1	1	1	1
	Adj SS	193,286	0.023	55.51	41,850	167.296	13.14	2352	3.407	693.81
	Adj MS	193,286	0.023	55.51	41,850	167.296	13.14	2352	3.407	693.81
	*F*-value	56.55	0.01	12.73	12.24	74.07	3.01	0.69	1.51	159.17
	*p*-value	0.001	0.924	0.016	0.017	0.000	0.143	0.445	0.274	0.000

* smaller is better; ** larger is better.

**Table 3:** Composition of the dermal patch models, their thickness (*n* = 10), and moisture content (*n* = 3). Different letters indicate statistically significant difference (*p* < 0.05).

Model	Polymers *	Plasticizers **	Transferosomes	Thickness	Moisture Content
	%, *w*/*v*	%, *v*/*v*	mg Na-Humate	µm ± SD	% ± SD
DP-Contr	5	30	-	70.25 ± 1.10 ^a^	8.87 ± 1.93 ^a^
DP-LC	5	30	70	74.60 ± 1.56 ^b^	10.96 ± 1.95 ^a^
DP-HC	5	30	140	80.25 ± 2.53 ^c^	13.23 ± 1.59 ^a^

* Sodium alginate: hydroxypropyl methylcellulose ratio 1:1; ** Glycerin: propylene glycol ratio 1:2.

**Table 4:** Distribution of the number of samples in the groups according to their respective histopathological score.

		Score		
Group	1–3	4–6	7–9	10–12
DP-Contr	5	3	-	-
Reference product	-	2	6	-
DP-LC	-	-	1	7
DP-HC	-	4	4	-

## Data Availability

The original contributions presented in this study are included in the article. Further inquiries can be directed to the corresponding author.

## Abbreviations

The following abbreviations are used in this manuscript:

DEE	drug-entrapment efficiency
HPMC	hydroxypropyl methylcellulose
PBS	phosphate-buffered saline
PDI	polydispersity index
SD	standard deviation
SEM	scanning electron microscopy
TEM	transmission electron microscopy
TGF-*β*	transforming growth factor-*β*
